# The sleep and circadian modulation of neural reward pathways: a protocol for a pair of systematic reviews

**DOI:** 10.1186/s13643-017-0631-3

**Published:** 2017-12-02

**Authors:** Jamie E. M. Byrne, Greg Murray

**Affiliations:** Centre for Mental Health, Faculty Health, Arts and Design, Swinburne University of Technology, John St, Hawthorn, VIC 3122 Australia

**Keywords:** Sleep, Circadian rhythm, Diurnal rhythm, Time of day, Reward, systematic review

## Abstract

**Background:**

Animal research suggests that neural reward activation may be systematically modulated by sleep and circadian function. Whether humans also exhibit sleep and circadian modulation of neural reward pathways is unclear. This area is in need of further research, as it has implications for the involvement of sleep and circadian function in reward-related disorders. The aim of this paper is to describe the protocol for a pair of systematic literature reviews to synthesise existing literature related to (1) sleep and (2) circadian modulation of neural reward pathways in healthy human populations.

**Methods:**

A systematic review of relevant online databases (Scopus, PubMed, Web of Science, ProQuest, PsycINFO and EBSCOhost) will be conducted. Reference lists, relevant reviews and supplementary data will be searched for additional articles. Articles will be included if (a) they contain a sleep- or circadian-related predictor variable with a neural reward outcome variable, (b) use a functional magnetic resonance imaging protocol and (c) use human samples. Articles will be excluded if study participants had disorders known to affect the reward system. The articles will be screened by two independent authors. Two authors will complete the data extraction form, with two authors independently completing the quality assessment tool for the selected articles, with a consensus reached with a third author if needed. Narrative synthesis methods will be used to analyse the data.

**Discussion:**

The findings from this pair of systematic literature reviews will assist in the identification of the pathways involved in the sleep and circadian function modulation of neural reward in healthy individuals, with implications for disorders characterised by dysregulation in sleep, circadian rhythms and reward function.

**Systematic review registration:**

PROSPERO CRD42017064994

**Electronic supplementary material:**

The online version of this article (10.1186/s13643-017-0631-3) contains supplementary material, which is available to authorized users.

## Background

There is a growing interest in moderators of the human reward system, with biological rhythms (sleep and circadian function) being a particular focus because of their potential interplay in mental disorders. Sleep and circadian function have been shown to be important in numerous reward-related disorders (e.g. bipolar disorder, depression, drug and alcohol use; [1–8]) but is also relevant for optimal functioning in healthy individuals [9–11]. Remarkably, no review to date has systematically examined existing brain imaging evidence for sleep or circadian modulation of the neural pathways of reward. Understanding the neural pathways in these two relationships may illuminate new clinical targets for stabilising interacting biological rhythm and reward dysregulation in these disorders. This protocol will begin with a brief summary of the evidence for sleep and circadian modulation of neural reward pathways, indicate how imaging may examine neural reward, and suggest some important issues in operationalising reward, before turning to the methods and data analytic plan for the proposed systematic review.

There is substantial evidence from animal research that circadian and sleep function modulates neural reward pathways. For example, Sleipness, Sorg and Jansen [12, 13] demonstrated that time of day differences in dopamine transmission and reward-seeking behaviour was reliant on the central circadian pacemaker, the suprachiasmatic nucleus. Further, circadian clock gene expression in the core reward regions (ventral tegmental area [VTA], nucleus accumbens [NAc] and the medial and dorsolateral prefrontal cortex [mPFC, DLPFC]) all exhibit circadian rhythmicity [14, 15]. In relation to sleep, Hanlon, Andrzejewski, Harder, Kelley and Benca [16] found that depriving rats of rapid eye movement sleep led to a decrease in motivation to seek rewards the following day. In humans, Volkow et al. [17] found that sleep deprivation altered dopamine transmission in the ventral striatum, which may represent a pathway for the altered reward response seen in behavioural tasks [18–20] and positive mood response [21–23] following sleep perturbations.

In humans, neuroimaging methods, particularly commonly used functional magnetic resonance imaging (fMRI) paradigms, have potential to illuminate the putative sleep and circadian modulation of neural reward pathways. In fMRI, neural activity is inferred from changes in the blood-oxygen-level-dependent (BOLD) signal, premised on regional blood flow. A common approach to investigating neural reward functioning in fMRI is via presentation of reward stimuli within a task-based protocol [24]. Reward studies have typically used money, food, happy faces or attractive physical features to activate reward regions in fMRI [25]. Task-based fMRI contrasts with resting state fMRI, a paradigm of growing interest which aims to detect patterns of fluctuating synchronous connectivity between structurally distinct brain regions in the absence of a stimulus [26].

Any synthesis of fMRI studies of reward must attend to the multiple methodological dimensions on which studies may differ. One advantage of using task-based imaging is that it allows for event-related and block-based tasks to temporally distinguish between these reward components. Block designs employ blocks of similar trial types which give higher power across many trials; event-related designs can distinguish between trials in a block, and separate out components within a trial [27]. Reward in humans is probably not a unitary process. Event-related designs have the potential to distinguish between reward anticipation and reward consumption [28]. Block designs are less temporally sensitive, but more powerful in detecting effects [27–29]. A second methodological consideration is that imaging tasks use different stimuli (e.g. money, food, social rewards) to measure neural reward [25]. In the present context, sleep and circadian function may have different interactions with (for example) money and food, making it important to discriminate between studies using one or other of these reward stimuli. A final consideration is reward can be examined outside of the stimuli in resting state fMRI. Resting state fMRI may capture a physiological preparedness to engage with rewarding stimuli in the environment.

### Objectives

The aim of this study is to synthesise existing research on the putative modulation of neural reward pathways by sleep and circadian function. An initial scoping of the literature strongly suggested that (a) these two modulation propositions would be best investigated separately to minimise heterogeneity and facilitate synthesis, and (b) there were sufficient studies in each area to support two separate systematic reviews. Therefore, the objective of this study is to conduct a pair of systematic reviews, driven by the following questions.

### Research questions

1. What evidence is there for modulation of neural reward pathways by sleep?
2. What evidence is there for modulation of neural reward pathways by circadian function?

## Methods

This systematic review will be written using the Preferred Reporting Items for Systematic Reviews and Meta-Analysis Protocols (PRISMA-P) guidelines (Additional file 1). This systematic review has been registered with the International Prospective Register of Systematic Reviews (PROSPERO, registration number: CRD42017064994).

### Criteria for study inclusion

(i) Study methods. Studies will be included if they use naturalistic designs or experimental designs (including cross-sectional, longitudinal, case-control studies and cross-over) to manipulate sleep or circadian function to examine neural reward functioning through fMRI. In addition, articles must be written in English and published in a peer-reviewed journal.
(ii) Study participants. Studies will be eligible for inclusion if they include samples of human subjects of any age. In the sleep systematic review, studies that include insomnia, symptoms or disorders will be included if these quasi-experimental groupings formed the predictor variable, compared to another sleep comparison group. So too, in the circadian systematic review, delayed or advanced sleep-wake phase will be included if these symptoms or disorders are related to another circadian function comparison group. In both reviews, clinical samples with psychological or physical illnesses that may affect reward will be excluded. Studies using samples with neurological or physiological disorders that have a pronounced effect on sleep (e.g. narcolepsy, Kleine-Levin syndrome, obstructive sleep apnoea) or circadian function (e.g. blind individuals or traumatic brain injury) will be excluded from both reviews. Studies that use pharmacological, acupuncture, transmagnetic stimulation or administered reward-related substances (alcohol, drugs [including caffeine and nicotine]) will be excluded, as this intervention may affect both the sleep or circadian predictor variable and the neural reward activation outcome variable. Individuals who have diagnoses of disorders known to affect the reward system (e.g. bipolar disorder, major depressive disorder, gambling disorder, alcohol and drug use disorders) will be excluded. Studies investigating altered reward response specific to a population (e.g. displaying images of alcohol to heavy alcohol users) will also be excluded, on the grounds that they effectively add an additional interaction term to the two broad relationships of interest in this systematic review.
(iii) Search strategy for study identification. Relevant health and neuroscience electronic databases (Scopus, PubMed, Web of Science, ProQuest, PsycINFO and EBSCOhost) will be searched for articles from the database inception until October 2017. Reference lists of selected articles, relevant reviews and meta-analyses and supplementary data files will be examined to identify any additional articles. The search strategy includes examining the article title, abstracts and keywords for relevant criteria (Table 1).

**Table 1:** Example search for sleep modulation of neural reward using keywords in Scopus

1 TITLE-ABS-KEY(sleep OR *REM OR adenosine OR nap)
2 TITLE-ABS-KEY(striatum OR “anterior cingulate” OR “ventral tegment* OR putamen OR VTA OR NAc OR accumben* OR “medial prefrontal cortex” OR MPFC OR “orbitofrontal cortex” OR OFC OR thalamus OR insula OR “dorsolateral prefrontal cortex” OR DLPFC OR amygdal* OR “locus coeruleus” OR LC OR dopamine* OR seroton* OR limbic OR caudate)
3 TITLE-ABS-KEY (reward OR arousal OR happ* OR food OR money OR positiv* OR affect OR emoti* OR reinforcement OR instrumental)
4 TITLE-ABS-KEY(imag* OR FMRI OR MRI)
5 NOT TITLE-ABS-KEY(rat OR rodent OR mouse OR mice OR hamster OR drosophila)
6 (LIMIT-TO(DOCTYPE,“ar ”) OR LIMIT-TO(DOCTYPE,“ip”)
7 2 OR 3
8 1 AND 4 AND 5 AND 6 AND 7

As seen in Tables 1 and 2, search term 1 aims to identify studies collecting data on sleep (Table 1) or circadian function (Table 2). All studies must have a predictor variable which is an experimental manipulation, or a naturalistic (quasi-experimental) quantification of sleep or circadian function.

- For the sleep review, studies that included different sleep stages or insomnia symptoms will be included as well as more traditional sleep deprivation or sleep extension studies.
- For the circadian function review, studies will be deemed to speak to the circadian function of reward if they use a repeated-measure protocol at different times of day (measuring a diurnal [daily] rhythm) and measure circadian phase through preformed chronotype (morning, evening type individuals) groups or use more endogenous markers of circadian function such as circadian genes, melatonin or cortisol levels.

**Table 2:** Example search for circadian modulation of neural reward using keywords in Scopus

1 TITLE-ABS-KEY(SCN OR suprachiasmatic OR circadian OR diurnal OR “clock gene*” OR “time of day” OR cortisol OR melatonin OR owl OR lark)
2 TITLE-ABS-KEY(striatum OR “anterior cingulate” OR “ventral tegment* OR putamen OR VTA OR NAc OR accumben* OR “medial prefrontal cortex” OR MPFC OR “orbitofrontal cortex” OR OFC OR thalamus OR insula OR “dorsolateral prefrontal cortex” OR DLPFC OR amygdal* OR “locus coeruleus” OR LC OR dopamine* OR seroton* OR limbic OR caudate)
3 TITLE-ABS-KEY (reward OR arousal OR happ* OR food OR money OR positiv* OR affect OR emoti* OR reinforcement OR instrumental)
4 TITLE-ABS-KEY(imag* OR FMRI OR MRI)
5 NOT TITLE-ABS-KEY(rat OR rodent OR mouse OR mice OR hamster OR drosophila)
6 (LIMIT-TO(DOCTYPE,“ar”) OR LIMIT-TO(DOCTYPE,“ip”)
7 2 OR 3
8 1 AND 4 AND 5 AND 6 AND 7

Search term 2 identifies studies collecting data on the neural basis of reward, while search term 3 identifies reward stimuli that may be used in imaging studies. Neural reward activation as measured by fMRI will be the outcome variable of both the planned systematic literature reviews. Reward functioning can be examined as an outcome of reward tasks, or through resting state scans that examine brain regions or neuronal activity associated with rewards. Search terms will be broad, including terms for reward stimuli, neural reward regions of interest and whole-brain analyses in fMRI studies. Search term 4 identifies fMRI paradigms, with search term 5 excluding commonly used animals in sleep and circadian research, and limits to full articles that are already in press (search term 6). Search term 7 aims to identify studies that have searched for or identified neural reward regions or used an imaging paradigm with reward-based stimuli.

### Study selection

Articles will be screened in two stages. Stage 1 (titles and abstract) will screen for inclusion and exclusion criteria (see Additional file 2). Stage 2 will collate the study selection results, and two authors will review the full texts for any discrepancies with Cohen’s Kappa for inter-rater agreement assessed and reported. If agreement cannot be reached, a third reviewer from the authors will make the final decision.

### Data extraction

A data extraction form in Microsoft Excel will be used to compile the method and results from each of the selected studies for the (1) sleep systematic review and (2) the circadian systematic review (see Additional file 3). Two independent reviewers will complete this form for the selected articles. Given the diverse measurements of sleep, circadian function and reward, this form will detail the sleep or circadian variable and how this was used in the method, whether a stimuli was or was not used in the neuroimaging protocol (with a description of the reward stimuli), participant information and characteristics, the imaging procedures used and regions of interest investigated and/ or identified, results, interpretation and limitations of the research, with two reviewers independently completing this extraction. Two authors will then complete a quality assessment of the articles using the Effective Public Health Practice Project (http://www.ephpp.ca/PDF/Quality%20Assessment%20Tool_2010_2.pdf; [30]) quality assessment tool measuring the robustness of articles through an examination of selection bias, study design, confounders, blinding, data collection methods, withdrawal and dropout, intervention integrity and analyses. The strength of this measure is that it allows for component and global ratings to be made; thus, components in this literature that may be less relevant (such as blinding) can be discussed without losing the important cross-study evaluation of the relevant quality assessment components. Where consensus is not reached on the quality assessment, a third reviewer will be relied on. In studies which investigate bi-directional influences between sleep/circadian and reward activation, only the primary direction of interest here (sleep and circadian modulation of reward) will be considered.

### Data analysis

As we expect the studies to be heterogeneous in both the sleep and circadian systematic reviews and the operationalisation of reward variables, the main data analysis will follow a narrative synthesis of articles in both cases. Popay et al.’s [31] four-step framework for developing an effective narrative synthesis will be used. The first step in the narrative synthesis will provide an explanation for how sleep or circadian function may causally impact different neural reward pathways. This step will map the potential neural pathways of reward in humans, and how these may relate to the different reward stages (reward anticipation and reward outcome). The second step entails a preliminary synthesis of the identified studies, describing the pattern of results by sleep and circadian function separately. The primary aim of this step will be to identify whether the neural reward outcome is modulated by sleep or circadian function, paying attention to the region affected, the direction of effect and the magnitude of observed effects in each individual study. A third step will involve analysis of these findings across studies, concentrating on cross-study design and methodological factors that may explain discrepancies between studies. For the present review, an important focus at this step will be cross-study differences in reward measurement (stimuli and imaging method used) and the reward stage (anticipation and outcome) examined. The final step will involve assessing the strength of evidence for the sleep and circadian modulation of reward as organised by reward stage and region. This will involve collating the results of the EPHP quality assessment (above) to see if some findings should be weighted more highly through both the quantity of evidence for an approach and the design qualities which may best address the current research question for the final narrative synthesis.

## Discussion

Currently, no systematic literature reviews have considered the impact of either sleep or circadian function on neural reward pathways. This is an important gap in knowledge, because recent empirical and theoretical evidence (e.g. [32–36]) have suggested a disturbed interaction between sleep/circadian function, and reward processes may be pivotal to serious reward-related psychopathologies including bipolar disorder, major depression and alcohol and drug use disorders. Better evidence in the general population for the putative existence, direction and strength of such relationships at the neural level in a pair of systematic reviews has the potential to improve understanding and ultimately management of a range of psychopathologies.

### Limitations

We expect that the primary limitation of these systematic literature reviews will be heterogeneity across identified studies. The measurement of neural reward activation has not been standardised across studies, with different designs using different types of reward stimuli, and varied focus between reward stages (anticipation and outcome). In addition, some studies will investigate neural reward through stimuli-response paradigms while others use resting state data to speak to a neural preparedness to engage in reward behaviour. While all these studies may speak to a sleep and circadian modulation of neural reward activation, differences in study paradigms will be important qualifications on any synthesis. On the basis of the results of the narrative synthesis, however, we expect to be able to identify which protocols may best illuminate the sleep and circadian modulation of neural reward activation in future research.

A final potential limitation of the literature is that few studies are expected to speak to both sleep and circadian function, which are known to be deeply interdependent. As such, this pair of reviews will emphasise that while it is scientifically important to examine sleep and circadian functioning separately, practically, these two studies will be confounded by the respective sleep or circadian process. As a result, these two systematic reviews can help inform future research ofsome of the ways that neuroimaging may better manage the interplay between sleep and circadian rhythms in their joint determination of neural reward functioning.

## Additional files

Additional file 1: PRISMA-P (Preferred Reporting Items for Systematic review and Meta-Analysis Protocols) 2015 checklist: recommended items to address in a systematic review protocol*. (DOC 60 kb)

Additional file 2: Article Selection. (XLSX 8 kb)

Additional file 3: Data Extraction. (XLSX 11 kb)
